# Supplementary material for: Decline of physical activity in early adolescence: A 3-year cohort study
Source: PLoS One. 2020 Mar 11;15(3):e0229305. doi: 10.1371/journal.pone.0229305 (PMC7065740; doi:10.1371/journal.pone.0229305)
Supplement: S1 Table — (DOCX) [file pone.0229305.s001.docx]

**S1 Table. Independent samples T-test comparison of physical fitness and somatic characteristics between boys from the total sample who were excluded (n=120) and the ones who were included (n=81) in the initial sample at age 11.**

| Measurement | Status | Mean | SD | t | df | Sig. |
| --- | --- | --- | --- | --- | --- | --- |
| Standing broad jump (cm) | excluded | 160.16 | 21.62 | -0.20 | 190 | 0.845 |
|  | included | 160.80 | 22.33 |  |  |  |
| Obstacle course backwards (s) | excluded | 16.55 | 5.56 | 0.68 | 189 | 0.496 |
|  | included | 16.01 | 4.67 |  |  |  |
| 20-s drumming test (repetitions) | excluded | 13.06 | 3.78 | -0.85 | 178 | 0.395 |
|  | included | 13.56 | 3.79 |  |  |  |
| Flamingo balance test (trials/min) | excluded | 17.45 | 7.75 | 1.22 | 189 | 0.224 |
|  | included | 16.04 | 7.49 |  |  |  |
| Sit and reach (cm) | excluded | 15.82 | 6.48 | -2.03 | 190 | 0.043 |
|  | included | 17.80 | 6.53 |  |  |  |
| Shoulder circumduction (cm) | excluded | 75.23 | 34.12 | -1.23 | 189 | 0.222 |
|  | included | 81.54 | 34.18 |  |  |  |
| Handgrip strength (kg) | excluded | 21.79 | 4.03 | 1.19 | 189 | 0.237 |
|  | included | 21.04 | 4.37 |  |  |  |
| Bent arm hang (s) | excluded | 23.55 | 21.36 | -0.70 | 189 | 0.483 |
|  | included | 25.94 | 24.68 |  |  |  |
| 20-m shuttle run (cumulative of laps) | excluded | 53.80 | 21.76 | -0.84 | 183 | 0.402 |
|  | included | 56.78 | 25.55 |  |  |  |
| Heigt (cm) | excluded | 150.09 | 7.09 | 0.57 | 193 | 0.568 |
|  | included | 149.47 | 7.75 |  |  |  |
| Weight (kg) | excluded | 44.39 | 11.35 | -0.34 | 193 | 0.737 |
|  | included | 44.98 | 12.14 |  |  |  |
| Triceps skinfold (mm) | excluded | 14.29 | 6.68 | 0.08 | 189 | 0.935 |
|  | included | 14.21 | 6.17 |  |  |  |
| Biceps skinfold (mm) | excluded | 8.81 | 4.79 | 0.07 | 189 | 0.944 |
|  | included | 8.76 | 4.69 |  |  |  |
| Subscapular skinfold (mm) | excluded | 10.28 | 7.22 | -0.33 | 189 | 0.740 |
|  | included | 10.64 | 7.28 |  |  |  |
| Suprailiac skinfold (mm) | excluded | 12.93 | 8.36 | -0.50 | 189 | 0.615 |
|  | included | 13.60 | 9.56 |  |  |  |
| Elbow breadth (cm) | excluded | 5.99 | 0.49 | 0.73 | 189 | 0.465 |
|  | included | 5.94 | 0.49 |  |  |  |
| Wrist breadth (cm) | excluded | 4.93 | 0.37 | 0.46 | 188 | 0.647 |
|  | included | 4.90 | 0.40 |  |  |  |
| Calf circumference (cm) | excluded | 31.30 | 3.66 | -0.05 | 189 | 0.960 |
|  | included | 31.33 | 3.54 |  |  |  |
| Mid-thigh circumference (cm) | excluded | 43.07 | 5.52 | -0.16 | 190 | 0.874 |
|  | included | 43.18 | 5.57 |  |  |  |
| Arm length (cm) | excluded | 66.72 | 3.86 | -0.06 | 189 | 0.956 |
|  | included | 66.75 | 4.70 |  |  |  |
| Leg length (cm) | excluded | 87.39 | 4.88 | 0.89 | 189 | 0.376 |
|  | included | 86.71 | 5.47 |  |  |  |
| Shoulder breadth (cm) | excluded | 32.43 | 1.99 | -0.10 | 189 | 0.924 |
|  | included | 32.46 | 2.82 |  |  |  |
| Pelvic breadth (cm) | excluded | 23.42 | 2.34 | -0.53 | 189 | 0.596 |
|  | included | 23.61 | 2.31 |  |  |  |
| Femoral breadth (cm) | excluded | 8.98 | 0.58 | 0.87 | 189 | 0.384 |
|  | included | 8.90 | 0.59 |  |  |  |
| Ankle breadth (cm) | excluded | 6.90 | 0.41 | -0.35 | 189 | 0.729 |
|  | included | 6.92 | 0.44 |  |  |  |
