# Supplementary material for: Decline of physical activity in early adolescence: A 3-year cohort study
Source: PLoS One. 2020 Mar 11;15(3):e0229305. doi: 10.1371/journal.pone.0229305 (PMC7065740; doi:10.1371/journal.pone.0229305)
Supplement: S2 Table — (DOCX) [file pone.0229305.s002.docx]

**S2 Table. Independent samples T-test comparison of physical fitness and somatic characteristics between girls from the total sample who were excluded (n=105) and the ones who were included (n=79) in the initial sample at age 11.**

| Measurement | Status | Mean | SD | t | df | Sig. |
| --- | --- | --- | --- | --- | --- | --- |
| Standing broad jump (cm) | excluded | 155.05 | 21.77 | 0.95 | 176 | 0.345 |
|  | included | 151.78 | 23.42 |  |  |  |
| Obstacle course backwards (s) | excluded | 16.62 | 6.06 | -0.15 | 176 | 0.878 |
|  | included | 16.52 | 5.85 |  |  |  |
| 20-s drumming test (repetitions) | excluded | 15.21 | 3.52 | 0.17 | 163 | 0.863 |
|  | included | 15.12 | 3.17 |  |  |  |
| Flamingo balance test (trials/min) | excluded | 15.95 | 7.30 | 1.33 | 176 | 0.185 |
|  | included | 14.46 | 7.19 |  |  |  |
| Sit and reach (cm) | excluded | 22.19 | 7.26 | 0.33 | 177 | 0.745 |
|  | included | 21.84 | 6.57 |  |  |  |
| Shoulder circumduction (cm) | excluded | 76.16 | 27.46 | 1.69 | 177 | 0.093 |
|  | included | 68.55 | 32.15 |  |  |  |
| Handgrip strength (kg) | excluded | 20.75 | 4.01 | 0.62 | 177 | 0.534 |
|  | included | 20.38 | 3.85 |  |  |  |
| Bent arm hang (s) | excluded | 20.69 | 18.19 | -0.54 | 177 | 0.587 |
|  | included | 22.38 | 23.05 |  |  |  |
| 20-m shuttle run (cumulative of laps) | excluded | 49.43 | 17.79 | -0.01 | 180 | 0.996 |
|  | included | 49.44 | 18.07 |  |  |  |
| Heigt (cm) | excluded | 151.38 | 6.63 | 1.16 | 180 | 0.248 |
|  | included | 150.16 | 7.30 |  |  |  |
| Weight (kg) | excluded | 44.71 | 10.04 | 0.55 | 180 | 0.586 |
|  | included | 43.89 | 9.32 |  |  |  |
| Triceps skinfold (mm) | excluded | 14.83 | 5.67 | -0.67 | 180 | 0.502 |
|  | included | 15.43 | 6.22 |  |  |  |
| Biceps skinfold (mm) | excluded | 9.28 | 4.60 | -0.76 | 180 | 0.451 |
|  | included | 9.83 | 5.03 |  |  |  |
| Subscapular skinfold (mm) | excluded | 11.04 | 6.59 | -0.09 | 180 | 0.930 |
|  | included | 11.13 | 6.11 |  |  |  |
| Suprailiac skinfold (mm) | excluded | 14.88 | 7.98 | -0.70 | 180 | 0.483 |
|  | included | 15.77 | 8.93 |  |  |  |
| Elbow breadth (cm) | excluded | 5.76 | 0.32 | 0.52 | 180 | 0.603 |
|  | included | 5.74 | 0.33 |  |  |  |
| Wrist breadth (cm) | excluded | 4.81 | 0.29 | 0.56 | 180 | 0.577 |
|  | included | 4.75 | 0.27 |  |  |  |
| Calf circumference (cm) | excluded | 31.59 | 3.31 | 0.86 | 180 | 0.392 |
|  | included | 31.16 | 3.34 |  |  |  |
| Mid-thigh circumference (cm) | excluded | 44.04 | 5.26 | 0.37 | 180 | 0.713 |
|  | included | 43.75 | 4.93 |  |  |  |
| Arm length (cm) | excluded | 66.75 | 3.53 | 0.93 | 180 | 0.355 |
|  | included | 66.22 | 4.01 |  |  |  |
| Leg length (cm) | excluded | 87.17 | 4.13 | 2.27 | 180 | 0.024 |
|  | included | 85.55 | 5.45 |  |  |  |
| Shoulder breadth (cm) | excluded | 32.63 | 2.05 | 1.06 | 180 | 0.291 |
|  | included | 32.31 | 1.82 |  |  |  |
| Pelvic breadth (cm) | excluded | 23.62 | 1.94 | 0.11 | 180 | 0.915 |
|  | included | 23.59 | 1.83 |  |  |  |
| Femoral breadth (cm) | excluded | 8.56 | 0.50 | 0.68 | 180 | 0.498 |
|  | included | 8.51 | 0.47 |  |  |  |
| Ankle breadth (cm) | excluded | 6.46 | 0.36 | -0.18 | 180 | 0.855 |
|  | included | 6.47 | 0.33 |  |  |  |
