# Supplementary material for: Decline of physical activity in early adolescence: A 3-year cohort study
Source: PLoS One. 2020 Mar 11;15(3):e0229305. doi: 10.1371/journal.pone.0229305 (PMC7065740; doi:10.1371/journal.pone.0229305)
Supplement: S3 Table — (DOCX) [file pone.0229305.s003.docx]

**S3 Table. Independent samples T-test comparison of physical fitness and somatic characteristics between boys from the original sample who were excluded (n=84) and the ones who were included (n=21) in the final sample at age 14.**

| Measurement | Status | Mean | SD | t | df | Sig. |
| --- | --- | --- | --- | --- | --- | --- |
| Standing broad jump (cm) | excluded | 189.54 | 26.95 | 0.04 | 119 | 0.965 |
|  | included | 189.25 | 28.18 |  |  |  |
| Obstacle course backwards (s) | excluded | 132.62 | 38.92 | 0.15 | 118 | 0.882 |
|  | included | 131.20 | 38.90 |  |  |  |
| 20-s drumming test (repetitions) | excluded | 16.33 | 4.32 | -0.80 | 119 | 0.423 |
|  | included | 17.15 | 3.39 |  |  |  |
| Flamingo balance test (trials/min) | excluded | 18.00 | 19.93 | 0.67 | 119 | 0.503 |
|  | included | 14.95 | 7.94 |  |  |  |
| Sit and reach (cm) | excluded | 24.68 | 8.07 | -0.60 | 119 | 0.553 |
|  | included | 25.85 | 7.75 |  |  |  |
| Shoulder circumduction (cm) | excluded | 89.99 | 40.29 | 0.71 | 119 | 0.481 |
|  | included | 82.90 | 44.69 |  |  |  |
| Handgrip strength (kg) | excluded | 31.17 | 7.66 | 0.45 | 119 | 0.652 |
|  | included | 30.35 | 5.82 |  |  |  |
| Bent arm hang (s) | excluded | 37.05 | 34.00 | 0.49 | 119 | 0.625 |
|  | included | 33.15 | 23.14 |  |  |  |
| 20-m shuttle run (cumulative of laps) | excluded | 64.54 | 24.32 | -0.49 | 119 | 0.624 |
|  | included | 67.55 | 28.41 |  |  |  |
| Heigt (cm) | excluded | 170.72 | 9.05 | 0.59 | 120 | 0.560 |
|  | included | 169.45 | 7.97 |  |  |  |
| Weight (kg) | excluded | 61.80 | 12.56 | 0.26 | 120 | 0.792 |
|  | included | 61.01 | 10.40 |  |  |  |
| Triceps skinfold (mm) | excluded | 13.05 | 6.42 | 0.33 | 120 | 0.743 |
|  | included | 12.53 | 6.79 |  |  |  |
| Biceps skinfold (mm) | excluded | 7.08 | 3.92 | -0.45 | 120 | 0.652 |
|  | included | 7.54 | 5.19 |  |  |  |
| Subscapular skinfold (mm) | excluded | 9.71 | 5.13 | -0.06 | 120 | 0.949 |
|  | included | 9.79 | 4.82 |  |  |  |
| Suprailiac skinfold (mm) | excluded | 12.44 | 8.72 | -0.23 | 120 | 0.818 |
|  | included | 12.33 | 7.36 |  |  |  |
| Elbow breadth (cm) | excluded | 5.08 | 0.503 | 1.09 | 120 | 0.279 |
|  | included | 4.66 | 1.043 |  |  |  |
| Wrist breadth (cm) | excluded | 3.99 | 0.395 | 1.42 | 120 | 0.159 |
|  | included | 3.51 | 0.785 |  |  |  |
| Calf circumference (cm) | excluded | 36.40 | 3.604 | -0.36 | 120 | 0.720 |
|  | included | 30.86 | 6.899 |  |  |  |
| Mid-thigh circumference (cm) | excluded | 49.97 | 4.948 | 0.60 | 120 | 0.553 |
|  | included | 56.53 | 12.64 |  |  |  |
| Arm length (cm) | excluded | 76.97 | 3.89 | 0.09 | 120 | 0.926 |
|  | included | 76.88 | 4.51 |  |  |  |
| Leg length (cm) | excluded | 99.28 | 5.28 | 1.26 | 120 | 0.209 |
|  | included | 97.64 | 5.52 |  |  |  |
| Shoulder breadth (cm) | excluded | 36.87 | 2.59 | 0.27 | 120 | 0.791 |
|  | included | 36.71 | 2.41 |  |  |  |
| Pelvic breadth (cm) | excluded | 27.03 | 5.81 | 0.48 | 120 | 0.631 |
|  | included | 26.39 | 2.03 |  |  |  |
| Femoral breadth (cm) | excluded | 9.32 | 0.68 | 1.54 | 120 | 0.127 |
|  | included | 9.07 | 0.61 |  |  |  |
| Ankle breadth (cm) | excluded | 7.40 | 0.62 | -0.32 | 120 | 0.752 |
|  | included | 7.46 | 1.03 |  |  |  |
