# Supplementary material for: Decline of physical activity in early adolescence: A 3-year cohort study
Source: PLoS One. 2020 Mar 11;15(3):e0229305. doi: 10.1371/journal.pone.0229305 (PMC7065740; doi:10.1371/journal.pone.0229305)
Supplement: S4 Table — (DOCX) [file pone.0229305.s004.docx]

**S4 Table. Independent samples T-test comparison of physical fitness and somatic characteristics between girls from the original sample who were excluded (n=102) and the ones who were included (n=29) in the final sample at age 14.**

| Measurement | Status | Mean | SD | t | df | Sig. |
| --- | --- | --- | --- | --- | --- | --- |
| Standing broad jump (cm) | excluded | 165.20 | 24.49 | -0.51 | 111 | 0.614 |
|  | included | 167.77 | 21.76 |  |  |  |
| Obstacle course backwards (s) | excluded | 14.77 | 4.24 | 1.59 | 110 | 0.114 |
|  | included | 13.41 | 3.23 |  |  |  |
| 20-s drumming test (repetitions) | excluded | 18.42 | 3.59 | -1.96 | 112 | 0.053 |
|  | included | 19.97 | 4.09 |  |  |  |
| Flamingo balance test (trials/min) | excluded | 13.17 | 11.76 | 0.85 | 112 | 0.398 |
|  | included | 11.27 | 5.67 |  |  |  |
| Sit and reach (cm) | excluded | 32.33 | 8.96 | -1.26 | 111 | 0.212 |
|  | included | 34.63 | 7.62 |  |  |  |
| Shoulder circumduction (cm) | excluded | 94.44 | 78.88 | 0.31 | 112 | 0.758 |
|  | included | 89.83 | 33.31 |  |  |  |
| Handgrip strength (kg) | excluded | 27.19 | 5.10 | 0.72 | 112 | 0.475 |
|  | included | 26.40 | 5.40 |  |  |  |
| Bent arm hang (s) | excluded | 26.04 | 27.67 | -1.48 | 112 | 0.141 |
|  | included | 34.87 | 28.99 |  |  |  |
| 20-m shuttle run (cumulative of laps) | excluded | 41.60 | 19.05 | -1.44 | 108 | 0.134 |
|  | included | 47.30 | 16.90 |  |  |  |
| Heigt (cm) | excluded | 166.04 | 6.03 | 1.29 | 112 | 0.201 |
|  | included | 164.38 | 6.16 |  |  |  |
| Weight (kg) | excluded | 58.90 | 9.32 | 1.01 | 112 | 0.316 |
|  | included | 56.90 | 9.37 |  |  |  |
| Triceps skinfold (mm) | excluded | 17.36 | 5.64 | 0.52 | 112 | 0.607 |
|  | included | 16.74 | 5.59 |  |  |  |
| Biceps skinfold (mm) | excluded | 9.60 | 4.17 | 1.24 | 112 | 0.217 |
|  | included | 8.53 | 3.65 |  |  |  |
| Subscapular skinfold (mm) | excluded | 12.45 | 5.62 | 0.46 | 112 | 0.645 |
|  | included | 11.90 | 5.68 |  |  |  |
| Suprailiac skinfold (mm) | excluded | 19.09 | 7.93 | 0.17 | 112 | 0.865 |
|  | included | 18.80 | 7.96 |  |  |  |
| Elbow breadth (cm) | excluded | 5.78 | 0.47 | -0.93 | 112 | 0.354 |
|  | included | 5.87 | 0.35 |  |  |  |
| Wrist breadth (cm) | excluded | 4.88 | 0.28 | -1.52 | 112 | 0.131 |
|  | included | 4.97 | 0.26 |  |  |  |
| Calf circumference (cm) | excluded | 36.34 | 7.08 | 1.49 | 112 | 0.138 |
|  | included | 34.36 | 2.51 |  |  |  |
| Mid-thigh circumference (cm) | excluded | 50.22 | 5.40 | 0.95 | 112 | 0.345 |
|  | included | 49.17 | 4.71 |  |  |  |
| Arm length (cm) | excluded | 73.82 | 3.04 | -0.64 | 112 | 0.524 |
|  | included | 74.25 | 3.48 |  |  |  |
| Leg length (cm) | excluded | 95.69 | 11.80 | 1.14 | 112 | 0.258 |
|  | included | 93.14 | 5.52 |  |  |  |
| Shoulder breadth (cm) | excluded | 35.52 | 1.91 | 1.56 | 112 | 0.121 |
|  | included | 34.79 | 2.80 |  |  |  |
| Pelvic breadth (cm) | excluded | 26.37 | 1.76 | 0.06 | 112 | 0.950 |
|  | included | 26.34 | 1.68 |  |  |  |
| Femoral breadth (cm) | excluded | 8.41 | 0.72 | 0.19 | 112 | 0.849 |
|  | included | 8.38 | 0.71 |  |  |  |
| Ankle breadth (cm) | excluded | 6.60 | 0.31 | 0.28 | 112 | 0.782 |
|  | included | 6.58 | 0.33 |  |  |  |
