# Supplementary material for: Decline of physical activity in early adolescence: A 3-year cohort study
Source: PLoS One. 2020 Mar 11;15(3):e0229305. doi: 10.1371/journal.pone.0229305 (PMC7065740; doi:10.1371/journal.pone.0229305)
Supplement: Attachment — Submitted filename: PLOS one 3 year PA review Sep 2019.docx [file pone.0229305.s006.docx]

Oversell, the results of the study are relevant in determining PA levels of boys and girls in Slovenia. However, the paper requires some major modifications in terms of how the data is reported and interpreted, to ensure clarity to the reader. The discussion and conclusion should support the findings, without overstating or establishing any causal inference.

**Abstract**

Line 17: please change ‘The study’ to ‘This study’

Line 18: please change to ‘boys and girls aged 11 and 14’. Please state n = for each age. From this sentence, it is not clear if the 11 year olds and 14 year olds were the same children (i.e. you analyzed their PA at aged 11, and then followed up 3 years later by analyzing their PA levels again at aged 14). Please make this clear to the reader. Also, can you please state how long they wore the Bodymedia for (i.e. how many days), and if the measurements were taken at the same time of year (please provide month and date)

Line 20-21: It is not clear about when PA was measured, and what you mean by subsample at the age 11, and final subsample at age 11 and 14. Can you please re-word to clarify this for the reader

Line 21: please use abbreviation (MVPA)

Line 22: please clarify what you mean by ‘longitudinally measured’ and provide n =. What ages are you referring to? Both 11 and 14 years? Or just 11 years? And how did you compare?

Line 23: please add ‘a’ before representative, and delete ‘the’ before age 11

Line 24: please use abbreviation (MVPA)

Line 24 – 24: compared to what?

Lines 24-26: please provide p values. The results section is not clear for the reader. You have ES, the eta squared symbol, and p values. Can you please provide a concise statement about the results, and use consistent reporting values (i.e. p values, ES, t values, f ratios etc)

Line 28: please use abbreviation (MVPA)

Line 28-29: please use abbreviation (MVPA)

Lines 30-32: The conclusions do not match the aim or findings. Can you please reword to include an appropriate conclusion based on the findings, as you do not directly measure schoolwork, participation in PE lessons, or extracurricular PA/PA outside the school environment

**Introduction**

Line 35: please delete the full stop and ‘it’, and replace with ‘and’ is one of the …..

Line 41: please add ‘the’ before adult

Line 42: please insert a comma before which

Line 43: please write World Health Organization in full before abbreviating

Line 48: ‘suitable contents’ is not clear for the reader. Can you please define

Lines 49 – 52: is there any evidence that the guidelines should differentiate between childhood and adolescence? Could you please clarify what you mean by childhood and adolescence (i.e. provide the ages in years). Is there any evidence that you can provide to support increased sedentary behavior in adolescence?

Lines 52-54: can you please provide the specific ages of childhood and adolescence (see comment above), and also provide evidence to support why it is important to analyse these changes. What does the literature state in terms of the transition from childhood and adolescence? How does this impact PA in adulthood etc.

Lines 54-56: I am not sure that ‘the matter is further complicated’ in relation to your study, as you are not comparing children from different countries, only Slovenia. So I would delete ‘the matter is further complicated’. You also use the word ‘different’ three times in this sentence. Could you please reword to allow the sentence to flow better for the reader

Line 59: please provide evidence that the age groups you are referring to are the ‘most problematic’

Line 63 delete ‘the’ before recent

Paragraph two (lines 60 – 78): I am not sure on the relevance of the paragraph to your study. You discuss advantages and limitations of questionnaires, DLW and accelerometers (none of which are utilized in your study). But you do not mention wearable technologies. And then the paragraph suddenly finishes, and moves on to discuss PA in Slovenia, which does not flow. As you are not comparing these methods (i.e. questionnaires, DLW or accelerometers) in your study I would remove this paragraph, and include pertinent and relevant information on wearable technologies and the use of the Body Media Sense wear armband to assess PA in children

Line 79: please specify the ages of the school children

Lines 81-86: Here you define preadolescents, and puberty. However, in the first paragraph (lines 50-51) you talk about the difference between childhood and adolescence. As you are only looking at PA in children aged 11 and 14, I do not think you can refer to ‘issues/problems’ between childhood and adolescence (in the first paragraph), as you are not actually measuring this in your study. The world health organization defines adolescence as between the ages of 10 and 19. Early adolescence (i.e. puberty) may be defined as ages 10-14. I would suggest therefore, that you temper your introduction (particularly the first paragraph), as you are not really examining differences in PA between childhood and adolescence, but looking at the change in PA during early adolescence (i.e. puberty)

Line 81-82: please see comment above. Are you sure that 15-18 y is ‘preadolescent’? Or is this age group mid adolescence? My understanding is that 10-14 y is ‘preadolescent’.

Lines 83-91: This information would be better suited to the methodology section. It is also not clear from these sentences exactly how you will control for biological maturity. What do you mean by discriminating factor? How will you control for this in the analyses? Please state this clearly in the methodology section only (i.e. PHV) was entered as a covariate

Can you please provide a hypothesis. And also state how long you will measure PA for. You mention ‘longitudinally’. But do you just mean you will analyze their PA once when they are 11, and once again when they are 14. How long will you analyse PA for?

**Methods**

Line 96. What is ACDSi. Please define this abbreviation

Line 98. What do you mean by ‘multiple location approach’. Was their random stratified sampling from each area of the country?

Line 103. How were the subsample of n = 160 chosen? Was their random stratified sampling of the 385 children eligible?

Line 107: please delete of before 14

Line 108-109: how did you define sufficient wear time?

Line 128: delete ‘also’ before in

Line 129: why was a 7 d period chosen? Due to logistical reasons (i.e. amount of kit)? Does 7 d provide a reliable and valid measure of PA in these children?

Lines 132-134. What is this cut off based on? Why 5 d? and why > 90%?

Line 148: To have worn the devices for 7 d should the collection not have bene the following Wednesday? Or am I missing something

Lines 166 – 172. It is a little difficult to follow as a reader, who did not conduct the study, what you mean by first to second round of PA. Is this from October 2013 (i.e. aged 11), to October 2016 (i.e. aged 14)? What do you mean ‘final subsample’, and ‘initial subsample’, and ‘total sample’. Please be very clear and specific about the time (i.e. month/year), number and ages of children, sex etc that you are referring to

Line 171: please insert ‘the’ before following

Line 181: please state which variables, rather than just ‘multiple variables’

Line 182: provide n for ‘total sample’, provide n = for subsample, provide n = for subsample at age 11. I found it difficult to read this sentence, and to follow exactly which samples/children you are referring to.

Lines 182-187 may be more useful in line 165

Line 188: please state that SPSS was used in the first sentence, prior to stating a t-test was conducted

Can you please state how data is presented (e.g. mean and SD), and how effect sizes were calculated

**Results:**

Lines 198-200. Again this sentence is difficult to follow as a reader. Are you just repeating what you have mentioned in the methods section? Please be very clear and specific about the time (i.e. month/year), number and ages of children, sex etc that you are referring to. I would delete lines 198-200

Line 199: insert ‘the’ before subsample

Line 201. Please define ‘groups’

Line 205: please subscript the degrees of freedom

Line 208: can you please provide a citation to support his

Table 1: can you please removed the borders in the middle of the table, and only include borders at the top and bottom. Can you please state what you mean by average (was this the mean). Please include the mean and SD in one column only. Please include the minimum and maximum in once column only. Is there a reason why you are reporting both min-max and mean (SD)? Please use the SI units for age (i.e. y)

Line 215: when you state sig more PA. Do you mean MVPA was higher at age 11 compared to age 14? Or are you talking about total EE? Please format the parentheses appropriately and include the ES in the same parenthesis. Is this eta squared ES?

Please include ES for all analyses

Please be consistent with your terminology. You state on line 217 the ‘difference in MVPA’. Whereas again on line 218 you refer to ‘more active’. As you are not measuring overall PA, but looking at MVPA minutes, please use MVPA consistently, and remove any reference to ‘more physical activity’. I cannot see any reference to total EE. Is this what you mean by ‘more physically active’ – please use consistent terminology relevant to your outcome measures

Line 221 – 222.: please delete this sentence. Please make the results concise and clear. You have already described the analyses conducted in the analyses section, so you do not need to repeat again how the analyses was conducted

Line 222: Delete independent sample t test, and just sate what was found

Line 227: p<0.5 would not be significant. Is this a typing error?

Lines 227-229: can you please include the eta square value in the same parenthesis

Line 232: please delete

**Discussion**

Line 237: please delete the word critical, as we cannot determine causal inference that it is indeed critical. By PA do you mean MVPA or TEE?

Line 239: please delete the space between 7 and %. Can you please discuses the implications of an average of 40 min reduction in MVPA for girls aged 11 – 14, and a 10 min reduction for boys. Please discuss those who had very low MVPA (I.E. 24 or 18 min). What % of 11 and 14 year old boys and girls met the PA recommendations for Slovenia? The SD are rather large – suggesting a great deal of variance in the PA levels. Can you postulate why this is. Do you have any information on which schools these children came from, what areas they live in, their socioeconomic background etc?

Lines 264-269: This sentence is very long. Can you please re-word to make it easier for the reader to follow, as numerous thoughts and points are raised in this sentence.

Line 273: what do you mean by more efficient than girls of the same age in 1990?

Lines 276-277. I am not sure you can make such a confident statement given you did not directly measure any changes in physical fitness

Lines 278-280: Can you please reword this sentence. I do not understand what you mean by ‘the question arises as to how big a drop in PA levels from childhood does influence the physical efficiency of children when they reach adolescence’. What do you mean by efficiency? And children compared to adolescence? You only measured MVPA and total EE in 11 and 14 year olds.

Lines 281-284: as you did not directly measure PA and PE lessons, or support from friends etc, can you please reword/temper these sentences and relate directly to your findings.

Overall, the discussion needs some work in terms of the flow of sentences, and the tempering/relevance of the findings (see comments above on reporting total EE, discussing if differences are meaningful, reporting % of children that met PA guidelines etc). You cannot make any causal inference for some of the points you make (with regard to PE lesson, maturity etc), as you only looked at 7 d of MVPA at age 11 and age 14. Please ensure the discussion only directly relates to your findings. And any postulations are interpreted with caution.

Line 294-295. Please reword. It is not clear that you are discussing the wear time of the SWA in the present study compared to previous research.

Lines 300-301. Can you make this sentence more concise. You mention consistent measurement equipment and criteria separately, objective EE assessment. Are you simply referring to using the SWA with the PHV. Please use specific and consistent terminology, and do not overstate the measurements. Please provide the reference for the SWA being valid and reliable for MVPA and EE use over 7 d in children. With regards to the limitations, please also acknowledge only analyzing the PA for one week. You do not mention any data collection in terms of PE lesson, outside school PA or exercise, illness etc which may have been important for interpreting the findings

Line 321. Please insert pre-adolescents. Please replace similarly with similar

Line 323-324. You cannot determine causal inference from puberty (i..e PHV) and MVPA. It was only inserted as the covariate. It may not have been the reason why girls decreased their MVPA. I would suggest that future research needs to explore (quantitatively and qualitatively) the reasons why girls are decreasing their MVPA in Slovenia.

Lines 326 – 330. Can you please temper and/or remove these sentences. As they are not measured or related to the outcomes in your study. Please ensure the conclusion is relevant to the discussion and results
