# Supplementary material for: Decline of physical activity in early adolescence: A 3-year cohort study
Source: PLoS One. 2020 Mar 11;15(3):e0229305. doi: 10.1371/journal.pone.0229305 (PMC7065740; doi:10.1371/journal.pone.0229305)
Supplement: Attachment — Submitted filename: review comments.docx [file pone.0229305.s007.docx]

I reviewed an interesting manuscript titled “Decline of physical activity from childhood to adolescence: A 3-year longitudinal study” by Dr. Sember et al. The study investigated the changes in physical activity during 3y follow-up period in adolescents and showed interestingly that MVPA declined in girls but not in boys.

**General comments**

Although the introduction describes important areas related to this study, it does not adequately describe the available evidence on the decline of physical activity from previous studies. The Authors also bring some ideas that maturation is important in study of changes in physical activity, but do not explain why and what is the evidence. Please revise the introduction to better cover previous evidence and to explain what is needed and what gaps this study fills.

The Authors used a physical activity monitor that was used continuously trough 24 hours. Why the Authors only study MVPA and not sedentary behaviour and sleep. That would strengthen the paper significantly.

**Specific comments**

L79-81. The children in Slovenia are one of the most active paediatric populations. Please define how this was measured.

L95-115. A lot of children dropped off the study How representative this sample was regarding available characteristics such as socioeconomic status, body composition and size (e.g. BMI percentile/sd-score, body fat percentage). Please define these information here and not just in the results.

L127-128. Can the Authors define how SWA was validated. Was it validated against doubly labelled water? What was the agreement between methods. Please expand this information.

L134-135. Please comment the epoch length. Can the Authors show that 60 s epoch length can capture physical activity level reliably. Some studies have shown that 60 s epoch is not able to capture true levels of moderate and vigorous physical activity (1).

L158-163. The Authors need to put more emphasis in the validity of their physical activity data. For example, it is important to describe whether adult derived MET definition (3.5 mL/kg/min) was used or was more appropriate definition used. There are strong data suggesting that adult derived METs are not suitable to used in children (2). Furthermore, METs can produce a significant bias in physical activity assessment (3) and fixed MET thresholds for moderate and vigorous physical activity underestimate true prevalence of physical activity in overweigh and low fit individuals (4). Therefore, I suggest that the Authors carefully describe the procedure used in the study, consider their effect on the results, and highlight these in the limitations.

It seems that the Authors have data on 20 metre shuttle run test. Although it is not the most valid method to assess cardiorespiratory fitness especially during puberty, it could be used to assess maximal endurance capacity in adolescents. Can the Authors use those results to individually calibrate physical activity intensity? This would be important next step in physical activity research.

Some of these limitations are partly covered in the limitations section, but these needs to be expanded.

L163-165. Please describe which formula was used to calculate maturity offset? Mirwald or Moore? Please also describe the whole formula and its validity in this age group.

L182-183. Please describe Mirwald method earlier. Please also note that age at peak height velocity is not equal to onset of puberty which is mostly assessed as transition from stage 1 to stage 2 in stages described by Tanner. Please revise the terminology accordingly.

L192-194. How maturity was used in the model as covariate? It was a bit unclear to me was it only baseline maturity or change in maturity. It is important also to control changes in maturity because at any age children and adolescents mature at different rates.

L193-195. It not clear here whether time x sex interaction were studied or how data was in fact analysed. A figure of results could make it clearer and show the trend of physical activity level and interactions in boys and girls.

L216. Please describe how ES was defined.

L126-232. Does this mean that the results did not change when maturity was adjusted for? It is still a bit unclear whether only baseline maturity was used as a covariate. It would be important to know whether larger changes in maturity (i.e. faster rate of maturation) are related to for example larger decline in physical activity especially in girls. Furthermore, be also aware when interpreting the data using METs that changes in body composition during pubertal development are different in males and females with increased body adiposity in females and decreased adiposity in males. These changes may have an influence on MET-based estimates of physical activity as MET itself is adiposity confounded metric.

L237-239. Although merely speculative, is it possible that the decline of physical activity in girls is just because of increasing adiposity as suggested by Kujala et al. (4). Thus, are the same fixed intensity thresholds valid in assessment of physical activity intensity especially during youth when body size and composition as well as physical fitness change remarkably. Please consider these issues at least in limitations.

L248-258. As the Authors state school is probably important factor influencing physical activity and sedentary time in youth. I suggest that the Authors consider also biological explanations explained for example in the article by Eisenmann and Wickel (5).

L262-264. The Authors state that there were no differences in physical activity at the age of 11 and 14 after adjustment for maturity. I was wondering that wasn’t this the case also without the adjustment. Please clarify. Please also consider my other comments on maturity.

L271-274. Please describe what the Authors mean by physical efficiency.

1.	Gao Y, Melin M, Mäkäräinen K, Rantalainen T, Pesola AJ, Laukkanen A, et al. Children’s physical activity and sedentary time compared using assessments of accelerometry counts and muscle activity level. PeerJ. 2018 Aug 21;6:e5437.

2.	Saint-Maurice PF, Kim Y, Welk GJ, Gaesser GA. Kids are not little adults: what MET threshold captures sedentary behavior in children? Eur J Appl Physiol. 2016 Jan;116(1):29–38.

3.	Tompuri TT. Metabolic equivalents of task are confounded by adiposity, which disturbs objective measurement of physical activity. Front Physiol [Internet]. 2015 Aug 11 [cited 2019 Oct 3];6. Available from: http://journal.frontiersin.org/Article/10.3389/fphys.2015.00226/abstract

4.	Kujala UM, Pietilä J, Myllymäki T, Mutikainen S, Föhr T, Korhonen I, et al. Physical Activity: Absolute Intensity versus Relative-to-Fitness-Level Volumes. Med Sci Sports Exerc. 2017 Mar;49(3):474.

5.	Eisenmann J, Wickel E. The Biological Basis of Physical Activity in Children: Revisited. Pediatr Exerc Sci. 2009 Aug 1;21:257–72.
